# Spherical Polar Pattern Matching for Star Identification

**DOI:** 10.3390/s25134201

**Published:** 2025-07-05

**Authors:** Jingneng Fu, Ling Lin, Qiang Li

**Affiliations:** 1 Institute of Optics and Electronics, Chinese Academy of Sciences, Chengdu 610209, China; linling@ioe.ac.cn (L.L.); liqiang@ioe.ac.cn (Q.L.); 2 University of Chinese Academy of Sciences, Beijing 100049, China; 3 Youth Innovation Promotion Association, Chinese Academy of Sciences, Beijing 100049, China

**Keywords:** star sensor, all-sky star identification, star pair identification, spherical polar pattern, relative azimuth histogram

## Abstract

To endow a star sensor with strong robustness, low algorithm complexity, and a small database, this paper proposes an all-sky star identification algorithm based on spherical polar pattern matching. The proposed algorithm consists of three main steps. First, the guide star is rotated to be a polar star, and the polar and azimuth angles of neighboring stars are used as polar pattern elements of the guide star. Then, the relative azimuth histogram is applied to the spherical polar pattern matching, and a star pair after spherical polar pattern matching is identified through angular distance cross-verification. Finally, a reference star image is generated from the identified star pair to complete the matching process of all guide stars in the field of view. The proposed algorithm is verified by simulation experiments. The simulation results show that for a star sensor with a medium field of view (15° × 15°, 1024 × 1024 pixel) and a limiting magnitude of 6.0 Mv, the required database size is 161 KB. When false and missing star spots account for 50% of the guide stars and the star spot extraction error is 1.0 pixel, the average star identification time is 0.35 ms (@i7-4790), and the identification probability is 99.9%. However, when false and missing star spots account for 100% of the guide stars and the star spot extraction error is 5.0 pixel, the average star identification time is less than 2.0 ms, and the identification probability is 97.1%.

## 1. Introduction

Star sensors [1,2,3] play a crucial role in high-precision navigation tasks for spacecraft and denote core optical sensors in spacecraft attitude control systems. Using a celestial sphere as a reference coordinate system, star sensors can sense multiple stars and obtain their direction vectors in an image coordinate system. After identifying the stars, the corresponding direction vectors in the celestial coordinate system can be calculated, determining the attitude of the spacecraft. Recently, star identification has become a core technology in celestial navigation.

Star identification can be traditionally classified into subgraph isomorphism and pattern identification tasks [4]. Since the literal meanings of the two tasks are not mutually exclusive, this classification can cause confusion in understanding the classification process. In view of that, this study abandons the above classification method and divides star identification into two major categories according to the star pattern element: distributed and centralized star identification. 

In the distributed patterns, the stars involved have an equal status. The classic distributed pattern is triangular [5], and a triangular algorithm directly uses the angular distances between three stars as patterns and adopts a Hash table named k-vector [6,7] for rapid retrieval. Since the pattern dimension is only three, it contains many redundant matches, which can result in a low identification probability of a single triangle. Subsequently, polygon algorithms, which add stars on the triangle to increase the pattern dimension, have been developed [8,9,10]. However, due to the combinatorial explosion, these algorithms have high complexity and require a large database. By contrast, centralized patterns have multiple stars distributed around one [11,12,13,14,15,16] or more [17,18,19] main stars. Due to the high-dimensional centralized pattern, these algorithms have stronger robustness, which is currently the mainstream requirement in the field of star identification. The centralized patterns with one main star include the grid identification algorithm [11,12], the Log-Polar algorithm [13], the radial and dynamic cyclic pattern algorithm [14], the radial pattern voting algorithm [15], and the maximum radial pattern matching algorithm [16]. The grid identification algorithm sets the main star as the center of the image coordinate system and selects the nearest neighboring star as the zero position of the azimuth; therefore, the performance of the grid algorithm depends on the nearest star. The Log-Polar algorithm calculates the logarithm of the radial direction using the visual characteristics of the human eye [20]; this process shows no essential difference compared to the grid identification method. The radial and dynamic cyclic pattern algorithm defines an angle of the neighboring radial edges as a dynamic cyclic pattern to avoid the selection of the zero position of the azimuth. In this algorithm, a false or missing neighboring star will generate at least two false or missing cyclic pattern elements; thus, the radial and dynamic cyclic pattern algorithm has a low robustness. In the radial pattern voting algorithm, each star spot to be recognized requires local and global radial pattern votes, which increases computational complexity. However, the maximum radial pattern matching method abandons cyclic pattern matching. Instead, it first performs single-star matching using a radial pattern, and then cross-validates the angular distance of the main star pair. This algorithm has the advantages of low algorithm complexity and a small database, but it is applicable only to star sensors with well-calibrated photometry and intrinsic parameters; also, this algorithm has low robustness. The centralized patterns with two main stars include the star pair axial image template algorithm [17] and the star pair grid algorithm [18]. These algorithms avoid the selection of the zero position of the azimuth, and the number of pattern elements is the square of the guide star number, which results in a large database. The centralized pattern algorithms for more than two main stars [19] are used only in academic research because of the combinatorial explosion. In addition, deep learning has promoted the development of star identification, such as the star identification using convolutional neural networks [21,22] and graph neural networks [23]. Compared with traditional algorithms, deep learning-based algorithms have advantages in terms of universality. However, deep learning-based algorithms face the problems of large databases or high hardware requirements. In summary, star identification faces mutual constraints regarding robustness, complexity, and database capacity, which pose additional challenges to the star identification process.

To address the aforementioned challenges, this study proposes an all-sky star identification method based on spherical polar pattern matching, which uses relative azimuth histograms. The proposed algorithm includes three main steps. First, the guide star is rotated to become a polar star, and the polar and azimuth angles of neighboring stars are defined as polar pattern elements of the guide star. Then, the relative azimuth histogram is used for spherical polar pattern matching, and a star pair after spherical polar pattern matching is identified through angular distance cross-validation. Finally, a reference star image is generated from the identified star pair to complete the matching process of all guide stars in the field of view. According to the characteristics of the proposed algorithm, which first performs the spherical polar pattern matching (SPPM) and then angular distance testing (ADT), the proposed algorithm is named SPPM-ADT.

The rest of this paper is organized as follows. Section 2 describes the proposed star identification model in detail. Section 3 introduces the steps of the star identification process. Section 4 presents the simulation results and compares the proposed algorithm with related existing algorithms. Section 5 concludes this work and outlines future work directions.

## 2. Star Identification Model

### 2.1. Database

Guide stars with high brightness are less affected by noise and are more likely to be detected. When the number of guide stars in the field of view reaches a certain value, further increases in the number of guide stars will not improve the attitude measurement accuracy of a star sensor but will reduce the star identification efficiency. Due to the two mentioned reasons, this study selects the stars with a brightness greater than the limiting magnitude ${Mv}_{Max}$ of a star sensor to generate guide stars. 

The specific steps are as follows:

**Step 1**: Initialize a guide star set $\Omega_{GS}=\emptyset$;

**Step 2**: Set the attitude of a star sensor. Select $N_{SS}^{\left(G\right)}$ brightest stars whose angle with the optical axis does not exceed $\theta_{Max}$;

**Step 3**: Traverse all possible attitudes, repeat Step 2, and update the guide star set $\Omega_{GS}$. Finally, obtain $N_{GS}=\#\Omega_{GS}$ guide stars, where # represents the number of elements in the guide star set.

In this study, $\theta_{Max}$ represents the maximum polar angle, and due to its complex relationship with a star sensor’s parameters, there has been no analyzed model for the optimal selection of the $\theta_{Max}$ value. After determining the other parameters, this study determines the optimal value of $\theta_{Max}$ based on the maximum star identification probability.

The database includes the guide star region hash table, celestial coordinate table, brightness ranking hash table, spherical polar pattern table, and spherical polar pattern hash table, which are described below.

(1) Region Hash Table

The key of the region hash table is the celestial sphere region number, whose value is the number of all guide stars, and it is not greater than the celestial sphere region serial number.

First, the celestial sphere [−90°, + 90°] × [0°, 360°] is partitioned into equal intervals of ${∆}_{\alpha \delta }\times {∆}_{\alpha \delta }$, which can be expressed as follows:

(1) $${∆}_{\alpha \delta }=min\left\{∆\in Z\left|mod\left(180,∆\right)=0,∆\geq {∆}_{Min}\right.\right\}$$

where mod represents the modulo operation, and ${∆}_{Min}$ is the minimum coverage radius of the field of view. 

After division, the celestial sphere is partitioned into $N_{\delta }\times N_{\alpha }=\frac{180}{{∆}_{\alpha \delta }}\times \frac{360}{{∆}_{\alpha \delta }}$ parts, and then, the region number where the guide star is located is defined as follows:

(2) $$I=\left(\left\lceil \frac{\alpha }{{∆}_{\alpha \delta }}\right\rceil -1\right)N_{\delta }+\left\lceil \frac{90+\delta }{{∆}_{\alpha \delta }}\right\rceil$$

where a set $\left(\alpha ,\delta \right)$ denotes the celestial coordinates of the guide star, and ⌈⌉ is the ceiling function.

Finally, according to the region hash table and the optical axis direction, neighboring guide stars are obtained. This study assumes that the optical axis direction coordinates of a star sensor are $\left(\alpha_{o},\delta_{o}\right)$. Then, when the optical axis points to the low-latitude region $D_{\alpha }<180{}^{\circ}$ of the celestial sphere, where $D_{\alpha }=\frac{2\theta_{Max}}{cos\left(\left|\delta_{o}\right|+\theta_{Max}\right)}$, the region number set of the relevant guide stars is defined as follows:

(3) $$\left\{I=N_{\delta }mod\left(i-1,N_{\alpha }\right)+j\left|\left\lceil \frac{\alpha_{o}-R_{\alpha }}{{∆}_{\alpha \delta }}\right\rceil \leq i\leq \left\lceil \frac{\alpha_{o}+R_{\alpha }}{{∆}_{\alpha \delta }}\right\rceil ,\left\lceil \frac{90+\delta_{o}-\theta_{Max}}{{∆}_{\alpha \delta }}\right\rceil \leq j\leq \left\lceil \frac{90+\delta_{o}+\theta_{Max}}{{∆}_{\alpha \delta }}\right\rceil \right.\right\}.$$

However, when the optical axis points to the high-latitude region of the celestial sphere $D_{\alpha }\geq 180{}^{\circ}$, and the region number set where the relevant guide stars are located is defined by:

(4) $$\left\{I=N_{\delta }\left(i-1\right)+j\left|1\leq i\leq N_{\alpha },1\leq j\leq N_{\delta },\left\lceil \frac{90+\delta_{o}-\theta_{Max}}{{∆}_{\alpha \delta }}\right\rceil \leq j\leq \left\lceil \frac{90+\delta_{o}+\theta_{Max}}{{∆}_{\alpha \delta }}\right\rceil \right.\right\};$$

(2) Celestial Coordinate Table

The celestial coordinates of a guide star include right ascension and declination. In this study, the address of the guide star coordinate table is retrieved through the region hash table.

(3) Spherical Polar Pattern Table

**Figure 1:**
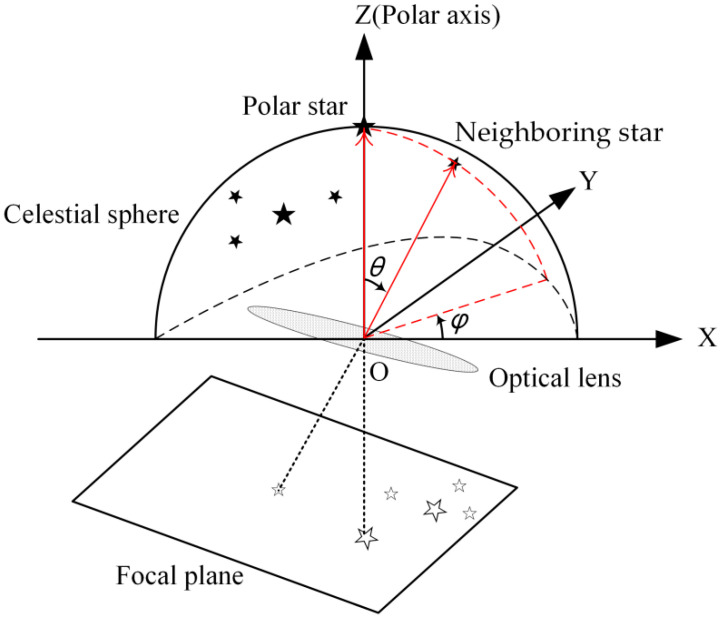
Illustration of the spherical polar pattern $\left(\theta ,\varphi \right)$ of a guide star.

The spherical polar pattern of a guide star is defined by a polar angle θ and an azimuth φ of its neighboring star in a celestial coordinate system, whose polar axis coincides with the guide star pointing, as shown in Figure 1.

The attitude matrix represented by the Euler angles 3-1-3 [24] is defined as follows:

(5) $$M_{313}\left(\alpha ,\delta ,\phi \right)=\left(\begin{array}{ccc} -sin\alpha cos\phi -cos\alpha sin\delta sin\phi \; & sin\alpha sin\phi -cos\alpha sin\delta cos\phi \; & cos\alpha cos\delta \;\\ cos\alpha cos\phi -sin\alpha sin\delta sin\phi \; & -cos\alpha sin\phi -sin\alpha sin\delta cos\phi \; & sin\alpha cos\delta \;\\ cos\delta sin\phi \; & cos\delta cos\phi \; & sin\delta \; \end{array}\right),$$

where $M_{313}\left(\alpha ,\delta ,\phi \right)$ indicates that the optical axis of the star sensor is pointing exactly to celestial coordinates $\left(\alpha ,\delta \right)$. 

In addition, it should be mentioned that the spherical polar pattern matching does not depend on the zero position of the azimuth; namely, a rotation angle ϕ can take any value. Therefore, it might be set that ϕ=0. Next, assume that the celestial coordinates of the mth guide star are $\left(\alpha_{m},\delta_{m}\right)$; then, the matrix for rotating the guide star to a polar star is expressed as follows:

(6) $$R_{m}^{\left(G\right)}=M_{313}^{-1}\left(\alpha_{m},\delta_{m},0\right)=\left(\begin{array}{ccc} -sin\alpha_{m} & cos\alpha_{m} & 0\\ -cos\alpha_{m}sin\delta_{m} & -sin\alpha_{m}sin\delta_{m} & cos\delta_{m}\\ cos\alpha_{m}cos\delta_{m} & sin\alpha_{m}cos\delta_{m} & sin\delta_{m} \end{array}\right).$$

If the celestial coordinates of the nth neighboring star are denoted by $\left(\alpha_{n},\delta_{n}\right)$, then its unit direction vector after rotation is expressed as follows:

(7) $$v_{mn}=R_{m}^{\left(G\right)}\cdot \left(\begin{array}{c} cos\delta_{n}cos\alpha_{n}\\ cos\delta_{n}sin\alpha_{n}\\ sin\delta_{n} \end{array}\right)=\left(\begin{array}{c} v_{mn1}\\ v_{mn2}\\ v_{mn3} \end{array}\right).$$

Afterward, in the celestial coordinate system with the mth star pointing as a polar axis, a polar angle $\theta_{mn}^{\left(G\right)}$ and an azimuth angle $\varphi_{mn}^{\left(G\right)}$ of the nth neighboring star are respectively expressed by:

(8) $$\theta_{mn}^{\left(G\right)}=arccos\left(v_{mn3}\right)=arccos\left(v_{m}^{T}\cdot v_{n}\right),$$

(9) $$\varphi_{mn}^{\left(G\right)}=\left\{\begin{array}{c} \arcsin\left(\frac{v_{mn2}}{\sqrt{v_{mn1}^{2}+v_{mn2}^{2}}}\right),\;\;\;\;\;\;\;\;\;\;\;v_{mn1}>0\;\mathrm{and}\;\;v_{mn2}>0,\\ 2\pi +\arcsin\left(\frac{v_{mn2}}{\sqrt{v_{mn1}^{2}+v_{mn2}^{2}}}\right),\;v_{mn1}>0\;\mathrm{and}\;\;v_{mn2}\leq 0,\\ \pi -\arcsin\left(\frac{v_{mn2}}{\sqrt{v_{mn1}^{2}+v_{mn2}^{2}}}\right),\;\;v_{mn1}\leq 0,\;\;\;\;\;\;\;\;\;\;\;\;\;\;\;\;\;\;\;\;\;\;\;\;\;\;\;\;\; \end{array}\right.$$

where the polar angle satisfies the condition of $0<\theta_{ij}^{\left(G\right)}\leq \theta_{Max}$, and the azimuth angle meets the condition of $0<\varphi_{ij}^{\left(G\right)}\leq 2\pi$.

Each row of the spherical polar pattern table corresponds to a guide star, with a length of $N_{PA}$ bytes. The spherical polar pattern of the mth star is denoted by $P_{m}^{\left(G\right)}=\left\{b_{m}^{\left(G\right)}\left[k\right]|1\leq k\leq N_{PA}\right\}$. Then, the bytes of $P_{m}^{\left(G\right)}$ are set according to the polar angle $\theta_{mn}^{\left(G\right)}$ and azimuth angle $\varphi_{mn}^{\left(G\right)}$ of the nth neighboring star with respect to the mth polar star, which can be expressed as follows:

(10) $$b_{m}^{\left(G\right)}\left[k\right]=\left\lceil \frac{\varphi_{mn}^{\left(G\right)}}{{∆}_{AZ}}\right\rceil ,k=\left\lceil \frac{\theta_{mn}^{\left(G\right)}}{{∆}_{PA}}\right\rceil ,$$

where ${∆}_{PA}$ and ${∆}_{AZ}$ are the quantization intervals of the polar and azimuth angles, respectively.

After processing the brightest stars, $N_{SS,m}^{\left(G\right)}\left(1\leq n\leq N_{SS,m}^{\left(G\right)}\right)$, within the maximum polar angle’s range, $\theta_{Max}={∆}_{PA}N_{PA}$, a spherical polar pattern $P_{m}^{\left(G\right)}$ of the mth guide star is obtained, where the upper limit of $N_{SS,m}^{\left(G\right)}$ is $N_{SS}^{\left(G\right)}$, that is, $N_{SS,m}^{\left(G\right)}\leq N_{SS}^{\left(G\right)}$.

Unlike the radial and dynamic cyclic pattern [14], a false or missing neighboring star can generate only a false or missing spherical polar pattern element.

Spherical polar pattern $P_{m}^{\left(G\right)}$ contains a large number of zero bytes, which results in a waste of storage space. Therefore, the spherical polar pattern should be compressed, and only non-zero pattern elements should be stored. Finally, each pattern element contains a pair consisting of a polar angle and an azimuth angle;

(4) Spherical Polar Pattern Hash Table

The number of polar pattern elements can vary among guide stars, so it is necessary to index the start and end addresses of the polar pattern elements of each guide star. The key of the spherical polar pattern hash table is the coordinate serial number of a guide star, and its value equals the total number of polar pattern elements of all the guide stars before the current one;

(5) Brightness Ranking Hash Table

The database of guide stars is arranged according to the celestial coordinates. However, for the spherical polar pattern matching in the all sky, guide stars with higher brightness have priority for matching, and guide stars are retrieved according to their brightness. The key of the brightness ranking hash table is the brightness serial number of a guide star, whose value is the coordinate serial number of the guide star.

### 2.2. Spherical Polar Pattern of Star Spots

The unit direction vector of the ith star spot in the image coordinate system is defined by:

(11) $$w_{i}=w\left(x_{i},y_{i}\right)={\left(w_{i1},w_{i2},w_{i3}\right)}^{T}=\frac{1}{\sqrt{{\left(x_{i}-x_{o}\right)}^{2}+{\left(y_{i}-x_{o}\right)}^{2}+f^{2}}}\left(\begin{array}{c} -\left(x_{i}-x_{o}\right)\\ -\left(y_{i}-y_{o}\right)\\ f \end{array}\right),$$

and the analogical celestial coordinates $\left(\alpha_{i},\delta_{i}\right)$ satisfy the following conditions

(12) $$\left\{\begin{array}{c} \sin\alpha_{i}=\frac{w_{i2}}{\sqrt{w_{i1}^{2}+w_{i2}^{2}}},\;\;\;\;\\ \cos\alpha_{i}=\frac{w_{i1}}{\sqrt{w_{i1}^{2}+w_{i2}^{2}}},\;\;\;\;\\ \sin\delta_{i}=w_{i3},\;\;\;\;\;\;\;\;\;\;\;\;\;\\ \cos\delta_{i}=\sqrt{1-w_{i3}^{2}}\;. \end{array}\right.$$

By substituting Equation (12) into Equation (6), the matrix for rotating the ith star spot to become a polar spot is defined by:

(13) $$R_{i}^{\left(S\right)}=\left(\begin{array}{ccc} -\frac{w_{i2}}{\sqrt{1-w_{i3}^{2}}} & \frac{w_{i1}}{\sqrt{1-w_{i3}^{2}}} & 0\\ -\frac{w_{i1}w_{i3}}{\sqrt{1-w_{i3}^{2}}} & -\frac{w_{i2}w_{i3}}{\sqrt{1-w_{i3}^{2}}} & \sqrt{1-w_{i3}^{2}}\\ w_{i1} & w_{i2} & w_{i3} \end{array}\right).$$

Further, assume that the unit direction vector of the jth neighboring star spot in the image coordinate system is $w_{j}$, then the unit direction vector after rotation can be expressed as follows:

(14) $$w_{ij}=R_{i}^{\left(S\right)}\cdot w_{j}=\left(\begin{array}{c} w_{ij1}\\ w_{ij2}\\ w_{ij3} \end{array}\right).$$

Afterward, in the celestial coordinate system where the pointing of the ith star spot denotes the polar axis, a polar angle $\theta_{ij}^{\left(S\right)}$ and an azimuth angle $\varphi_{ij}^{\left(S\right)}$ of the jth neighboring star spot are respectively expressed by:

(15) $$\theta_{ij}^{\left(S\right)}=arccos\left(w_{ij3}\right)=arccos\left(w_{i}^{T}\cdot w_{j}\right),$$

(16) $$\varphi_{ij}^{\left(S\right)}=\left\{\begin{array}{c} \arcsin\left(\frac{w_{ij2}}{\sqrt{w_{ij1}^{2}+w_{ij2}^{2}}}\right),\;\;\;\;\;\;\;\;\;\;\;w_{ij1}>0\;\mathrm{and}\;\;w_{ij2}>0,\\ 2\pi +\arcsin\left(\frac{w_{ij2}}{\sqrt{w_{ij1}^{2}+w_{ij2}^{2}}}\right),\;w_{ij1}>0\;\mathrm{and}\;\;w_{ij2}\leq 0,\\ \pi -\arcsin\left(\frac{w_{ij2}}{\sqrt{w_{ij1}^{2}+w_{ij2}^{2}}}\right),\;\;w_{ij1}\leq 0,\;\;\;\;\;\;\;\;\;\;\;\;\;\;\;\;\;\;\;\;\;\;\;\;\;\;\;\;\; \end{array}\right.$$

where the polar angle satisfies the condition of $0<\theta_{ij}^{\left(S\right)}\leq \theta_{Max}$, and the azimuth angle meets the condition of $0<\varphi_{ij}^{\left(S\right)}\leq 2\pi$.

The spherical polar pattern matching aims to reduce the influence of the star spot extraction error, denoted by $\sigma_{S}$. It should be noted that performing the compensation on the polar patterns of the guide and star spots is equivalent in computational aspect. Considering that the compensation is placed on the polar patterns of star spots rather than on the polar patterns of guide stars, there are at least two advantages:

(1) Reduce the polar pattern elements of the database;

(2) When the star spot extraction error changes, there is no need to update the polar pattern table of the database.

Set the bytes of pattern $P_{i}^{\left(S\right)}$ according to the polar angle matching probability of a single star spot as follows:

(17) $$b_{i}^{\left(S\right)}\left[k\right]=\left\lceil \frac{\varphi_{ij}^{\left(S\right)}}{{∆}_{AZ}}\right\rceil ,s^{*}<k\leq t^{*},$$

where the start and end positions are denoted by $\left(s^{*},t^{*}\right)={argmin}_{\left(s,t\right)}\left\{t-s\left|p\left(s,t\right)\right.\geq p_{Min},t,s\in N\right\}$, where $p_{Min}$ is the single-star polar angle matching probability threshold, and in this study, it is set to $p_{Min}=0.8$ according to the previous experience; $p\left(s,t\right)$ is the single-star polar angle matching probability, and $p\left(s,t\right)=\frac{1}{\sqrt{2\pi }\sigma_{\theta }}\int_{s{∆}_{PA}}^{t{∆}_{PA}}\exp\left(-\frac{{\left(\theta -\theta^{\left(S\right)}\right)}^{2}}{2\sigma_{\theta }^{2}}\right)d\theta$; $\sigma_{\theta }$ is the star pointing error, and $\sigma_{\theta }=\sigma_{S}/f$.

After processing the brightest neighboring star spots $N_{SS,i}^{\left(S\right)}\left(1\leq j\leq N_{SS,i}^{\left(S\right)}\right)$ in the maximum polar angle $\theta_{Max}$, the star spot polar pattern $P_{i}^{\left(S\right)}=\left\{b_{i}^{\left(S\right)}\left[k\right]|1\leq k\leq N_{PA}\right\}$ can be obtained. Due to the existence of false stars, the upper limit $N_{SS}^{\left(S\right)}$ of $N_{SS,i}^{\left(S\right)}$ is set to twice that of $N_{SS}^{\left(G\right)}$, that is, $N_{SS,i}^{\left(S\right)}\leq N_{SS}^{\left(S\right)}=2N_{SS}^{\left(G\right)}$.

On the one hand, the smaller the value of a polar angle quantization interval ${∆}_{PA}$ is, the sparser the spherical polar pattern $P_{i}^{\left(S\right)}$ and the lower the probability of mismatching will be. On the other hand, the larger the ${∆}_{PA}$ value is, the fewer the pattern matching times of $P_{i}^{\left(S\right)}$ and the smaller the computational amount will be. Thus, the optimal choice of ${∆}_{PA}^{*}$ is the minimum ${∆}_{PA}$ with constraints of t−s≤2 and $p\left(s,t\right)\geq p_{Min}$, which can be expressed as follows:

(18) $${∆}_{PA}^{*}={argmin}_{{∆}_{PA}}\left\{{min}_{\theta^{\left(S\right)}}\left\{p\left(s,t\right)\geq p_{Min},t-s\leq 2\right\}\right\},$$

where, argmin denotes the parameter value at which the optimized function attains its minimum value.

### 2.3. Relative Azimuth Histogram

**Figure 2:**
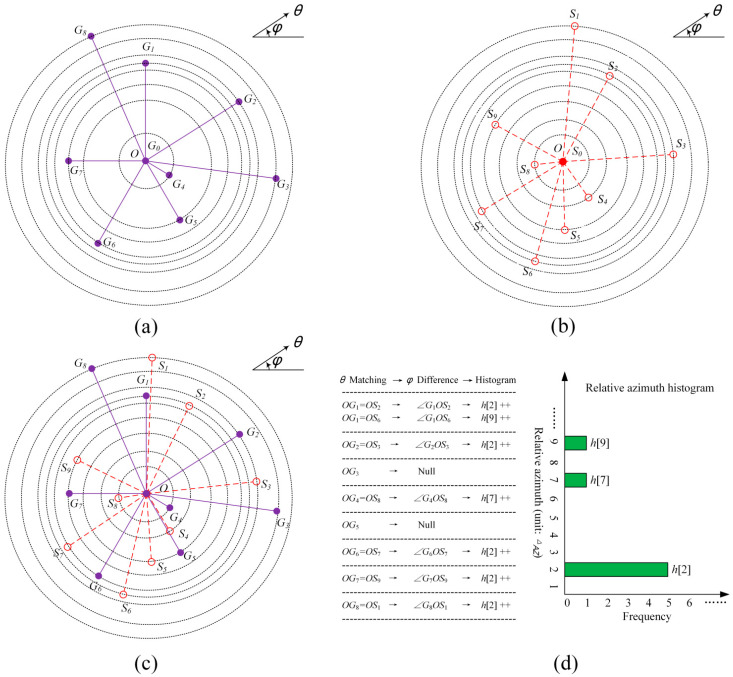
The calculation diagram of the relative azimuth histogram: (**a**) the spherical polar pattern of a guide star G_0_; (**b**) the spherical polar pattern of a star spot S_0_; (**c**) the pole O of the star spot coincides with that of the guide star; (**d**) at the same polar angle, the azimuths of the star spot and the guide star subtracted to calculate the relative azimuth histogram.

The spherical polar pattern elements of a guide star and the corresponding star spot have the same polar angle and fixed azimuth deviation. Due to the noise and quantization influence, the azimuth deviation is not unique, as shown in Figure 2. This study calculates the optimal azimuth deviation using a relative azimuth histogram.

Suppose the spherical polar pattern of the mth guide star is $P_{m}^{\left(G\right)}$, and the spherical polar pattern of the ith star spot in an image is $P_{i}^{\left(S\right)}$; then, the relative azimuth histogram of the mth guide star with respect to the ith star spot is defined as follows:

(19) $$h_{im}\left[s\right]=\#\left\{k\in \Omega_{PA,im}\left|mod\left(b_{i}^{\left(S\right)}\left[k\right]-b_{m}^{\left(G\right)}\left[k\right],N_{AZ}\right)=s\right.\right\},$$

where $\Omega_{PA,im}=\left\{k\left|b_{i}^{\left(S\right)}\left[k\right]>0,b_{m}^{\left(G\right)}\left[k\right]>0\right.\right\}$ is the polar angle matching set of the mth guide star and the ith star spot.

### 2.4. SPPM-ADT Algorithm

The guide star label corresponding to the maximum relative azimuth histogram matching is defined as follows:

(20) $$i^{*}={argmax}_{m}\left\{{max}_{s}\left\{h_{im}\left[s\right]+h_{im}\left[s+1\right]\right\}\right\}.$$

To overcome the influence of the azimuth quantization error, the neighboring histograms are summed in Equation (20).

Suppose the unit direction vector of the ith star spot in the image coordinate system and the corresponding guide star in the celestial coordinate system are denoted as $w_{i}$ and $v_{i^{*}}$, respectively. Accordingly, a label $j^{*}$ indicates the guide star with the optimal matching value of the jth star spot. The star pair identification is completed through angular distance cross-validation. First, the angular distances in an image coordinate system $\theta_{ij}^{\left(S\right)}={cos}^{-1}\left(w_{i}^{T}\cdot w_{j}\right)$ and a celestial sphere $\theta_{i^{*}j^{*}}^{\left(G\right)}={cos}^{-1}\left(v_{i^{*}}^{T}\cdot v_{j^{*}}\right)$ are calculated. If the condition of $\left|\theta_{ij}^{\left(S\right)}-\theta_{i^{*}j^{*}}^{\left(G\right)}\right|<3\sigma_{\theta }$ is met, then the star pair matching is completed, and the coarse attitude calculation is proceeded; otherwise, other star pairs are selected for matching. 

**Figure 3:**
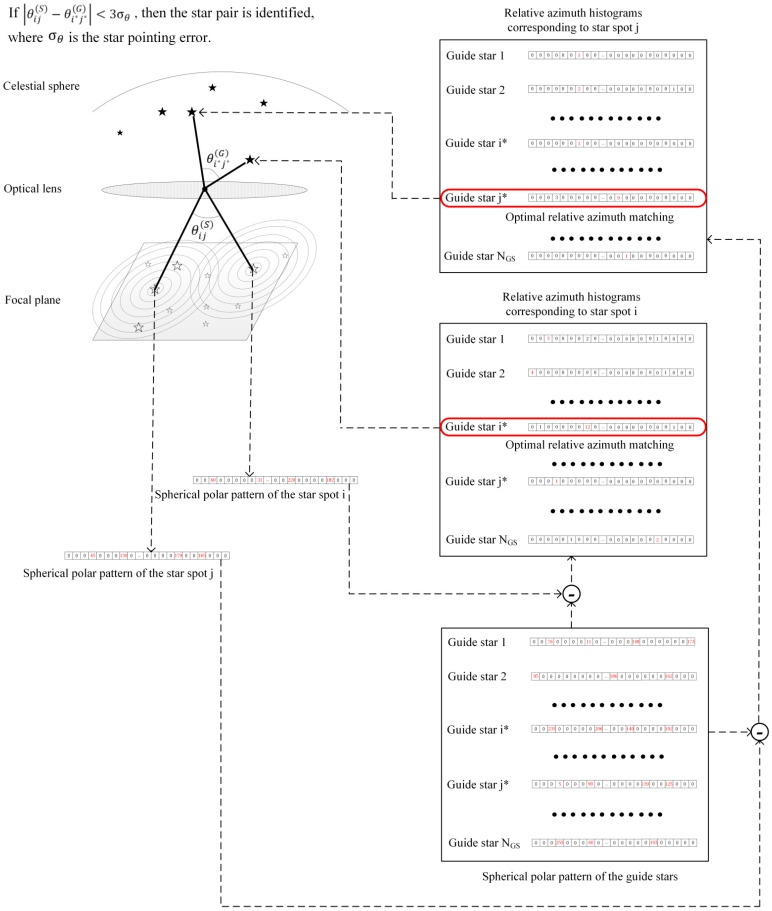
The SPPM-ADT diagram.

The calculation process of the SPPM-ADT algorithm is shown in Figure 3, where it can be seen that first the spherical polar pattern matching (SPPM) is performed, and then the angular distance testing (ADT) is conducted. Therefore, this algorithm is named SPPM-ADT. 

Finally, to reduce the complexity of the SPPM-ADT algorithms, this study presents the optimization details in programming:

(1) Convert the loop judgment given in Equation (19) into Boolean addition. Also, in addition to compressed polar pattern elements, the feature of the star spot should include a binary polar angle table;

(2) A polar angle matching number $\#\Omega_{PA,im}$ rarely exceeds the value of three and $\frac{1}{4}N_{SS,i}^{\left(S\right)}$. Most of the relative azimuth histograms obtained using Equation (19) are invalid. Accordingly, the initial value of the polar angle matching threshold is set to be a value larger than three and $\frac{1}{4}N_{SS,i}^{\left(S\right)}$;

(3) If the polar angle matching threshold is less than the azimuth histogram matching value ${max}_{s}\left\{h_{im}\left[s\right]+h_{im}\left[s+1\right]\right\}$, then the polar angle matching threshold is dynamically updated;

(4) For guide stars with the polar angle matching value smaller than the polar angle matching threshold, the relative azimuth histogram matching is terminated in advance;

(5) If the azimuth histogram matching value exceeds four, the corresponding guide star is very likely to be an optimal solution to Equation (20). For instance, for the estimated single-time relative azimuth histogram matching probability of 80%, the false alarm rate when the azimuth histogram matching value exceeds four is less than 5 × 10^−4^. Therefore, once the azimuth histogram matching value exceeds four, the matching guide star label is immediately output; the subsequent star spots are transferred to the azimuth histogram matching in the local celestial region, and the guide stars in the local celestial region are retrieved using the region hash table;

(6) Brighter guide stars and star spots have the priority in azimuth histogram matching; namely, subscripts i and m in Equation (20) are sorted according to the brightness level; a subscript *m* is retrieved from the brightness ranking hash table. 

After the above optimization steps, the identification time is decreased by two orders of magnitude.

## 3. Star Identification Steps

**Input:** The original star catalog, star image, a limit magnitude ${Mv}_{Max}$, the field of view FOV, star spot extraction error $\sigma_{S}$, magnitude random error $\sigma_{Mv}$;

**Output:** Star identification results.

The star identification steps are as follows:

**Step 1:** Generate the database;

**Step 2:** Extract the star spot coordinates $\left\{\left(x_{i},y_{i}\right)\right\}$ from the star image captured by a star sensor;

**Step 3:** Use the proposed SPPM-ADT algorithm for star pair matching. If a star pair is successfully matched, proceed to Step 4. If all star pairs in the field of view are traversed and the matching process cannot be completed, the star identification process fails, and the algorithm proceeds to Step 6;

**Step 4:** Use the star pair matching results and the singular value decomposition algorithm [25] to calculate the attitude matrix. Estimate the optical axis direction $\left(\alpha_{o},\delta_{o}\right)$ based on the attitude matrix. 

Combine the guide star region hash table and the guide star coordinate table to obtain the guide stars in the local celestial region. Generate a reference star image based on the celestial coordinates of the guide stars and attitude matrix. The estimated coordinates of a star spot are denoted by $\left({\hat{x}}_{j},{\hat{y}}_{j}\right)$, and if $\sqrt{{\left(x_{i}-{\hat{x}}_{j}\right)}^{2}+{\left(y_{i}-{\hat{y}}_{j}\right)}^{2}}<5\sigma_{S}$, then the ith star spot successfully matches the jth guide star. Next, traverse and match all star spots in the field of view using the above method;

**Step 5:** Generate the reference star image again based on the multi-star matching results and traverse and match all the star spots in the field of view using the same method as in Step 4. If the number of matched star spots exceeds four, the algorithm proceeds to Step 6; otherwise, the algorithm returns to Step 3;

**Step 6:** Output the matching results. 

As for the previous steps, Step 1 is performed offline and does not consume the star identification time. In Step 2, star spot extraction and image transmission are generally carried out synchronously, and this step is further divided into sub-steps of image segmentation, connected domain marking, star spot centroid calculation, and background parameter prediction. Step 3 employs the proposed star pair identification method of maximum spherical polar pattern matching. If the angular distance between stars is too small, Step 4 cannot generate an effective reference star image. In this study, the angular distance threshold between stars is set to 1/10 of the field of view according to experience. Steps 4 and 5 serve as a verification method for the star pair identification in Step 3 and can improve the attitude measurement accuracy of a star sensor.

## 4. Simulation Results Analysis and Discussion

This paper conducted simulation experiments on a computer equipped with 8 GB of memory and an Intel Core i7-4790 CPU, using Microsoft Visual C++ 6.0 to run a C program.

The attitude angle range of the star sensor was [−90°, 90°] × [0°, 360°] × [0°, 360°]. Considering the simulation time, one star image was generated every 2° × 2° × 2°, and a total of 90 × 180 × 180 = 2,916,000 star images were generated. 

To evaluate the performance of the star identification, the identification probability was defined as follows:

(21) $$p_{D}=\frac{The\;identification\;number\;of\;the\;star\;images}{The\;total\;number\;of\;star\;images}.$$

Similarly, the identification time was defined by:

(22) $$t_{D}=\frac{\sum_{i}The\;identification\;time\;of\;the\;ith\;star\;image}{The\;total\;number\;of\;star\;images}.$$

The simulation process was divided into two parts. First, the basic performance of the proposed SPPM-ADT on a star sensor with a medium field of view, whose parameters are shown in Table 1, was examined, and then, the proposed algorithm was compared with other similar algorithms.

### 4.1. Proposed Algorithm Performance Analysis

Based on the star sensor’s parameters in Table 1, the minimum coverage radius of the field of view was ${∆}_{Min}=\frac{FOV}{\sqrt{2}}=10.6{}^{\circ}$. Also, according to Equation (2), the celestial sphere division interval was ${∆}_{\alpha \delta }=12{}^{\circ}$, and the celestial sphere was divided into 450 parts. 

The azimuth angle satisfied the condition of 0<φ≤360°, and the number of azimuth quantization was $N_{AZ}=255$; the azimuth quantization interval was ${∆}_{AZ}=\frac{360{}^{\circ}}{255}$. According to Equation (18) and an upper limit of 5.0 pixel on the star spot extraction error, the polar angle quantization interval was ${∆}_{PA}=340^{\prime \prime }$. By optimizing the calculation process of the polar angle quantization number of $N_{PA}=87$, the maximum value of the corresponding polar angle was obtained as $\theta_{Max}=0.55FOV$.

This study selected stars with J2000 SAO [26] brighter than 9.0 Mv as the original star catalog, containing a total of 181,855 stars. By using the guide star generation method presented in Section 2.1, the known limiting magnitude of ${Mv}_{Max}=$ 6.0 Mv, the upper limit number of neighboring stars of $N_{SS}^{\left(G\right)}=15$, and the maximum polar angle of $\theta_{Max}=0.55FOV$ were calculated; after selection, 4224 guide stars were obtained, and the number of the spherical polar pattern elements of the guide stars was calculated as 56,449. Further, based on the above information, the data types and distribution of the database were obtained, as shown in Table 2, and the total size of the database was 160.7 KB.

To simulate the influence of the detector noise and test the robustness of the proposed star identification algorithm, this study set the star spot extraction error $\sigma_{S}$ to 0.1 pixel–5.0 pixel; the magnitude bias ($b_{Mv}$) was −0.5 Mv–0.5 Mv; and the random error ($\sigma_{Mv}$) was 0–1.0 Mv. Finally, the magnitude error was calculated by: $\sqrt{b_{Mv}^{2}+\sigma_{Mv}^{2}}$. 

To reduce the simulation workload, this study used the proposed SPPM-ADT method to calculate the results only for a single magnitude error, that is, $b_{Mv}=$ (−0.5–0.5) Mv and $\sigma_{Mv}=0$, and $b_{Mv}=0,\;{and\sigma }_{Mv}=\left(0–1.0\right)\;Mv$. For the magnitude error of a mixed type, the performance could be inferred from the above two cases.

**Figure 4:**
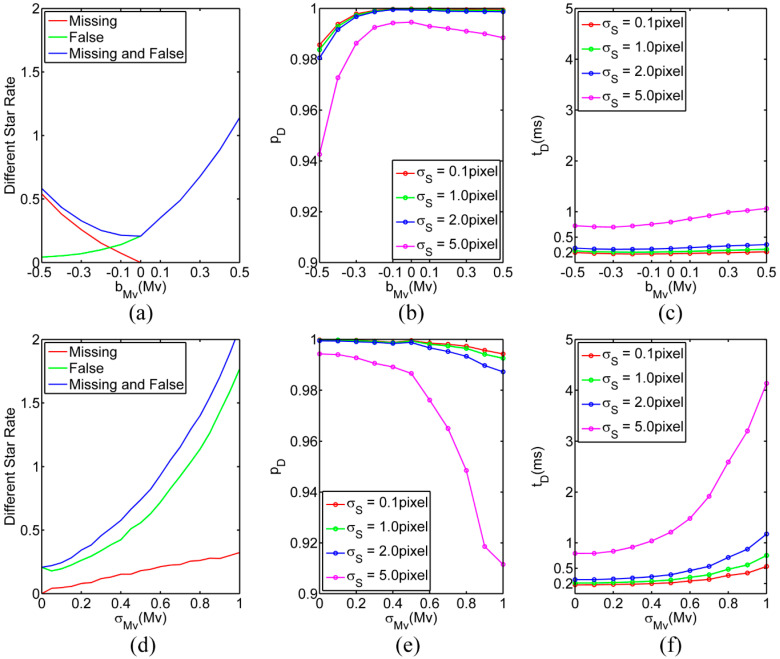
The results obtained for a magnitude bias of $b_{Mv}=$ (−0.5–0.5) Mv and a magnitude random error of $\sigma_{Mv}=$ 0: (**a**) the relative proportion of false and missing stars in the field of view to the guide stars; (**b**) the identification probability; (**c**) the identification time. The results obtained for a magnitude bias of $b_{Mv}=0$ and a magnitude random error of $\sigma_{Mv}=\;($0–1.0) Mv: (**d**) the relative proportion of false and missing stars in the field of view to the guide stars; (**e**) the identification probability; (**f**) the identification time.

First, the proportion of false and missing stars relative to the theoretically identifiable guide stars in the field of view was statistically analyzed, as shown in Figure 4a–d. For $b_{Mv}\;$= −0.45 Mv and $\sigma_{Mv}$ = 0, $b_{Mv}$= 0.2 Mv and $\sigma_{Mv}$ = 0, and $\sigma_{Mv}$ = 0.35 Mv and $b_{Mv}$ = 0, the proportion of false and missing stars in the field of view relative to the guide stars exceeded 50%. However, for $b_{Mv}$ = 0.45 Mv and $\sigma_{Mv}$ = 0, and $\sigma_{Mv}$ = 0.65 Mv and $b_{Mv}$ = 0, the proportion of false and missing stars in the field of view relative to the guide stars exceeded 100%.

After calibration of photometric and intrinsic parameters of the star sensor, the proportion of false and missing stars in the field of view relative to the guide stars was lower than 50% and the star spot extraction error was less than 1.0 pixel; also, the all-sky identification probability was higher than 99.95%, and the average identification time was shorter than 0.35 ms. In cases with inconsistent star catalog and working waveband of the star sensor, the magnitude error was large. When there were a large number of false and missing stars in the field of view relative to the guide stars or the intrinsic parameters were not calibrated, the star spot extraction error was also large. Namely, all these factors interfered with the all-sky star identification. For instance, when the proportion of false and missing stars in the field of view relative to the guide stars was close to 100% and the star spot extraction error was 5.0 pixel, the all-sky identification probability was approximately 97.19%, and the average identification time was 1.9 ms. However, for the same star spot extraction error of 5.0 pixel, when the proportion of false and missing stars in the field of view relative to the guide stars was close to 200%, the all-sky identification probability was only 91%. Thus, the all-sky star identification faced certain challenges, which could be one of the future research directions in the star identification method design.

In addition, after the guide stars and spherical polar pattern elements were determined, the identification probability and identification time did not change significantly with the limiting magnitude, as shown in Figure 4b,c. However, with the decrease in the limiting magnitude, the identification probability reduced significantly. Therefore, the limiting magnitude should be set with a positive deviation relative to the theoretical value.

Finally, sacrificing a small amount of star identification probability in exchange for less storage space and higher computational efficiency could be acceptable for star sensors with low-cost hardware deployment. The proposed SPPM–ADT algorithm set the upper limit of the number of neighboring stars involved in the pattern calculation of the guide star to $N_{SS}^{\left(G\right)}=9$. The number of guide stars (3096) was reduced by 1/4, and the number of the spherical polar pattern elements (27,219) was reduced by 1/2; then, the storage space of the database was only 90.2 KB. In addition, after calibration of photometric and intrinsic parameters of the low-cost star sensor, the star spot extraction error was less than 1.0 pixel, and the magnitude error was smaller than 0.5 Mv. Thus, the computational efficiency was increased by 15% while the all-sky identification probability remained as high as 99.55%, and the sacrificed star identification probability was lower than 0.5%.

### 4.2. Comparison with Other Algorithms

The comparison analysis with other algorithms faced two main challenges: (1) it was difficult to obtain the source codes of other star identification algorithms; and (2) the parameters of star sensors used in other studies differed from those used in this study. Namely, it should be noted that even if the same star identification algorithm is used with different star sensors, the optimal parameters of the algorithm differ, which introduces difficulties to the comparison process of different algorithms. To overcome this challenge and ensure a fair comparison of different algorithms, this study performed a one-to-one algorithm comparison, where the optimal parameters of the proposed SPPM-ADT algorithm were set according to the star sensor provided in a particular study used for the comparison. The comparison algorithms included the synthetic radial pattern matching (SRPM) algorithm [27], the recommended radial pattern matching algorithm (RRPM) [28], the radial triangle mapping matrix matching (RTMM) algorithm [19], and the angular distance matching score transform (ADMST) algorithm [29].

#### 4.2.1. Comparison Analysis of SPPM-ADT and SRPM Algorithms

According to the parameters of the star sensor used in [27], the parameters of the proposed SPPM-ADT were set as follows: the celestial sphere division interval was ${∆}_{\alpha \delta }=12{}^{\circ}$, and the celestial sphere was divided into 450 parts; the number of azimuth quantization was $N_{AZ}=255$, and the azimuth quantization interval was ${∆}_{AZ}=\frac{360{}^{\circ}}{255}$. Based on an upper limit of 8.0 pixel on the star spot extraction error, the polar angle quantization interval was ${∆}_{PA}=263^{\prime \prime }$. Further, by optimizing the number of polar angle quantization to $N_{PA}=115$, the maximum polar angle was calculated as $\theta_{Max}=0.58FOV$. The stars in the J2000 SAO catalog brighter than 7.0 Mv were used as the original star catalog, containing a total of 15,935 stars; this catalog was used to generate simulated star images. According to the limiting magnitude of the star sensor of ${Mv}_{Max}=$ 6.0 Mv, the upper limit number of neighboring stars was $N_{SS}^{\left(G\right)}=15$, and after star selection, 4141 guide stars were obtained; the number of spherical polar pattern elements of the guide stars was 56,927.

Based on the above parameters, the storage space occupied by the proposed SPPM-ADT algorithm could be calculated to be approximately 161 KB. In contrast, the database of the SRPM algorithm required a storage space of 661 KB, which was more than four times that of the SPPM-ADT.

In [27], a single-core Intel i5-3210M was used for simulation experiments. The single-core performance of the Intel i5-3210M is approximately 70% of that of the Intel i7-4790. In addition, excluding the hardware factors, the average identification time of the SRPM algorithm was still 20 times that of the proposed SPPM-ADT algorithm. Moreover, the robustness of the proposed SPPM-ADT algorithm was significantly better than that of the SRPM algorithm. For a star spot extraction error of 8.0 pixel and a magnitude random error of 0.4 Mv, and for a star spot extraction error of 2.5 pixel and a magnitude random error of 1.0 Mv, the star identification probability of the proposed SPPM-ADT algorithm was 10% higher than that of the SRPM algorithm, as shown in Table 3.

#### 4.2.2. Comparison Analysis of SPPM-ADT and RRPM Algorithms

Following the star sensor parameters in [28], the parameters of the proposed SPPM-ADT algorithm in this experiment were set as follows. The interval of celestial sphere division was ${∆}_{\alpha \delta }=9{}^{\circ}$, and the celestial sphere was divided into 800 parts. The number of azimuth quantization was $N_{AZ}=255$, and the azimuth quantization interval was ${∆}_{AZ}=\frac{360{}^{\circ}}{255}$. According to an upper limit of 4.0 pixel on the star spot extraction error, the polar angle quantization interval was ${∆}_{PA}=155^{\prime \prime }$. Further, by optimizing the number of polar angle quantizations to $N_{PA}=190$, the maximum polar angle was calculated to be $\theta_{Max}=0.48FOV$. Stars brighter than 7.5 Mv in the J2000 SAO catalog were used as the original star catalog, containing a total of 26,584 stars, which were used to generate simulated star images. According to the limiting magnitude of a star sensor of ${Mv}_{Max}=6.0\;Mv$, the upper limit number of neighboring stars was calculated as $N_{SS}^{\left(G\right)}=15$, 4279 guide stars were selected, and the number of the spherical polar pattern elements of the guide stars was 60,425. 

According to the above parameters, the proposed SPPM-ADT algorithm occupied approximately 170 KB of storage space, whereas the RRPM algorithm occupied approximately 776 KB of storage space, which was four times that of the SPPM-ADT algorithm.

Based on the hardware information provided in [28], a dual-core with a main frequency of 2.3 GHz was used in the verification process, so it could be inferred that a single-core Intel i3-2350M was used for the simulation experiment. The single-core performance of Intel i3-2350M is 50% of that of Intel i7-4790. Therefore, excluding the hardware factors, the average identification time of the RRPM algorithm was still five times that of the proposed SPPM-ADT algorithm. In addition, the robustness of the proposed SPPM-ADT algorithm was better than that of the RRPM algorithm. When the star spot extraction error was 1.0 pixel and the magnitude random error was 1.0 Mv, the star identification probability of the proposed SPPM-ADT was 99.89%, showing an improvement compared to that of the RRPM of 95.45%, as shown in Table 4.

#### 4.2.3. Comparison Analysis of SPPM-ADT and RTMM Algorithms

Based on the star sensor parameters used in [19], the parameters of the proposed SPPM-ADT were set as follows. The celestial sphere division interval was ${∆}_{\alpha \delta }=12{}^{\circ}$, and the celestial sphere was divided into 450 parts. The number of azimuth quantization was $N_{AZ}=255$, and the azimuth quantization interval was ${∆}_{AZ}=\frac{360{}^{\circ}}{255}$. According to an upper limit of 2.0 pixel on the star spot extraction error, the polar angle quantization interval was determined as ${∆}_{PA}=145^{\prime \prime }$. In addition, by optimizing the calculation value of the number of polar angle quantizations to $N_{PA}=218$, the corresponding maximum polar angle was obtained as $\theta_{Max}=0.55FOV$. Further, stars brighter than 7.5 Mv in the J2000 SAO catalog were used as the original star catalog, containing a total of 26,584 stars, which were used to generate simulated star images. According to the limiting magnitude of the star sensor of ${Mv}_{Max}=6.0Mv$, the upper limit number of neighboring stars was $N_{SS}^{\left(G\right)}=15$, and 3952 guide stars were selected; the number of the spherical polar pattern elements of the guide stars was 56,618.

According to the above settings, the storage space occupied by the proposed SPPM-ADT algorithm was calculated to be approximately 158 KB. In contrast, the database of the RTMM algorithm required a storage space of 2.73 MB, which was more than 15 times that of the proposed SPPM-ADT algorithm.

In [19], the authors used a single-core Intel i5-11600K for simulation experiments. The single-core performance of the Intel i5-11600K is 1.5 times that of the Intel i7-4790. However, the average identification time of the RTMM algorithm was still more than 10 times that of the proposed SPPM-ADT algorithm. In addition, the robustness of the proposed SPPM-ADT algorithm was better than that of the RTMM algorithm, as shown in Table 5.

#### 4.2.4. Comparison Analysis of SPPM-ADT and ADMST Algorithms

Using the parameters of the star sensor in [29], the parameters of the proposed SPPM-ADT algorithm were set as follows. The celestial sphere was divided into 288 parts with the celestial sphere division interval of ${∆}_{\alpha \delta }=15{}^{\circ}$. The number of azimuth quantization was $N_{AZ}=255$, and the azimuth quantization interval was ${∆}_{AZ}=\frac{360{}^{\circ}}{255}$. Based on an upper limit of 5.0 pixel on the star spot extraction error, the polar angle quantization interval was ${∆}_{PA}=193^{\prime \prime }$. Also, by optimizing the calculation value of the number of polar angle quantizations to $N_{PA}=146$, the maximum polar angle was obtained as $\theta_{Max}=0.46FOV$. The J2000 SAO stars brighter than 7.5 Mv were used as the original star catalog, containing a total of 26,584 stars, which were used to generate the simulated star images. According to the limiting magnitude of the star sensor of ${Mv}_{Max}=6.0\;Mv$, the upper limit number of neighboring stars was $N_{SS}^{\left(G\right)}=15$, 4401 guide stars were selected, and the number of the spherical polar pattern elements of the guide stars was 60,579.

According to the simulation setup, the storage space occupied by the SPPM-ADT algorithm was approximately 171 KB. The storage space required by the ADMST algorithm was not given in the original literature, but it was estimated based on 4931 stars containing 577,066 star pairs of guide stars. The storage space occupied by 3746 guide stars was calculated to exceed 1 MB, which was more than five times that of the proposed SPPM-ADT algorithm.

In [29], a single-core Intel i5-12490F was used for simulation experiments. The single-core performance of the Intel i5-12490F is approximately 1.5 times that of the Intel i7-4790. However, the average identification time of the ADMST algorithm was still more than 10 times that of the SPPMADT algorithm. The robustness of the proposed SPPM-ADT algorithm was better than that of the ADMST algorithm. When the star spot extraction error was 1.0 pixel and the magnitude random error was 1.5 Mv, the star identification probability of the SPPM-ADT algorithm was 97.62%, which was better than that of the ADMST algorithm of 96.0%, as shown in Table 6.

### 4.3. Discussion

In the above-presented comparison experiments, various star sensors with a medium field of view from 14.5° to 17° and a limiting magnitude of 6.0 Mv were used. For the proposed SPPM-ADT, the optimal solution of the maximum polar angle parameter $\theta_{Max}$ was around 0.5FOV, and it decreased with the field-of-view coverage area. Based on the principle of matching the maximum polar angle $\theta_{Max}$ with the field of view FOV, for a square field of view, the optimal solution of $\theta_{Max}$ would be 0.56FOV, and for a circular field of view, the optimal solution of $\theta_{Max}$ would be 0.50FOV. Therefore, for a star sensor with a sensitivity of 6.0 Mv, the optimal solution of the field of view was a square field of view of 15° × 15°, and a circular field of view of Φ16°.

Considering the experimental results provided in the related studies, the comparison algorithms can be sorted according to the star identification probability from high to low as follows: SPPM-ADT > ADMST > RTMM > RRPM > SRPM. According to the operation efficiency, the comparison algorithms can be sorted from high to low as follows: SPPM-ADT > RRPM > RTMM > ADMST > SRPM. According to the database storage space occupied from large to small, the algorithms can be sorted as follows: RTMM > ADMST > RRPM > SRPM > SPPM-ADT. 

Further, excluding the SPPM-ADT algorithm, the other comparison algorithms sacrificed the storage space occupied by the database to realize an efficient and highly robust star identification. The SPPM-ADT algorithm adopted retrieval and compression techniques, and by calculating the angular distance of star pairs online, it prevented the data storage of the angular distance like that of the ADMST and SRPM algorithms, so that the database only occupied a very small storage space. The data storage space required by the other algorithms was more than three times that of the proposed SPPM-ADT algorithm, and that of the SPPM-ADT algorithm did not exceed 180 KB.

The performance of the SRPM was significantly inferior to that of the other algorithms, demonstrating that if the pattern of the main star only included a radial pattern without a cyclic pattern, it would be insufficient to become a highly robust star identification algorithm.

Further, it was inferred that, due to the low-dimensional pattern of the database, the triangle, pyramid, and polygon algorithms could not be suitable for situations with large star spot extraction errors and many false and missing stars. However, the triangle, pyramid, and polygon algorithms had the advantage of rapid data retrieval due to a smaller search range when the star spot error was small (less than 0.1 pixel) and there were fewer guide stars in the field of view. After the spherical polar pattern elements were modified, the proposed SPPM-ADT algorithm could maintain the existing computational efficiency in the case of small star spot errors. The data type of the pattern elements was changed from unsigned char to unsigned short, and the range was changed from 0–255 to 0–65,535; accordingly, the database increased by 110 KB on the existing basis. However, the database of the proposed SPPM-ADT algorithm was still much smaller than that of the distributed method. Lastly, the modified SPPM-ADT algorithm was equivalent to distributed methods, such as the triangle, pyramid, and polygon algorithms. In this sense, the SPPM-ADT algorithm overcame the defect of the centralized algorithm in the matching of fewer guide stars in the field of view.

Similar to the studies used in the comparative analyses, the simulation experiments were initially performed in MATLAB, which is efficient in matrix operations but does not perform well for loop operations. Therefore, the MATLAB version of pattern matching should execute matrix operations rather than loop processing, and the polar pattern database should be stored in matrix form without compression. For the typical star sensor, such as that used in Section 4.1, in the experiments, the database size in the polar pattern matrix form was approximately 410 KB. Particularly, when the star spot error was small, the database size exceeded 4 MB, but the C version with the compressed polar pattern database did not exceed 280 KB. In addition, since the MATLAB version did not adopt loop pattern matching, the early termination matching strategy could not be efficiently implemented, which increased the probability of false matching, whereas the star identification probability dropped by approximately 2%. The coding of the MATLAB version was simpler than that of the C version, but the code of the C version could effectively compress the database and could be directly deployed on the star sensor hardware system. Thus, it could be concluded that it was worthwhile coding the proposed SPPM-ADT algorithm in the C language.

The above comparative experiments focused on using simulation data due to the following reasons: 

(1) Actual star images collected in real scenarios cannot cover the all-sky region, and actual star images used in related literature presenting different comparative algorithms vary and are commonly not public. Furthermore, since the comparison algorithm source codes are unavailable, comparative analysis using actual star images could be meaningless under given conditions;

(2) By contrast, the star catalog is publicly available, and simulation parameters and results can be easily obtained from the literature. Therefore, simulation-based comparisons between different algorithms could be considered more feasible and fairer.

**Figure 5:**
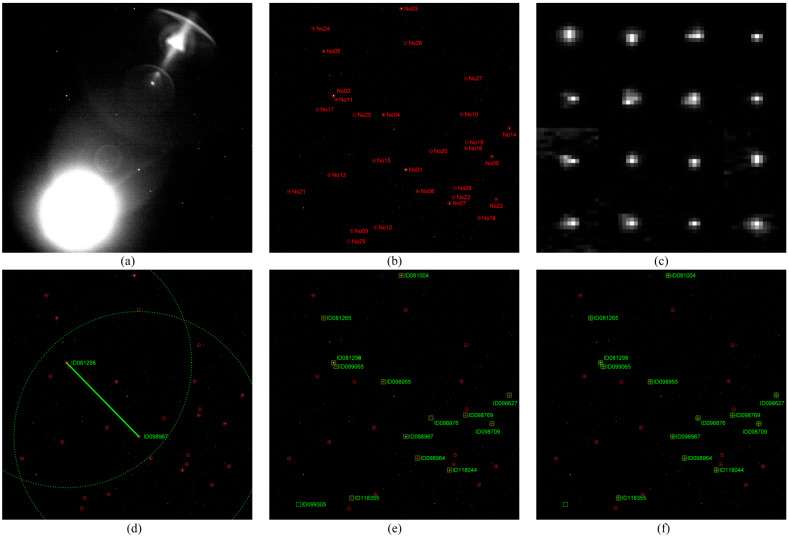
The processing steps of a star sensor equipped with the SPPM-ADT: (**a**) the Moon enters the field of view and forms a “ghost image”; (**b**) the star spots detected by the FMBBF [30], sorted from brightest to darkest, numbered as No01–No29, and marked with red circles; (**c**) morphologies of star spots No01–No16; (**d**) the results of star pair identification performed using the SPPM-ADT, where the arcs denote the neighboring star selection ranges for two main stars, and the IDXXX is the star address in SAO catalog; (**e**) the projections of the stars in the local celestial sphere onto the image plane obtained after the star pair identification, marked with green boxes; (**f**) star map recognition results obtained using nearest neighbor matching, marked with green crosshairs.

However, simulation experiments cannot replace real experiments. In addition, star sensors operating in orbit typically face the following challenges:

(1) When a star sensor passes through the South Atlantic Anomaly (SAA), many false star spots that cannot be distinguished in a single image frame can appear. In severe cases, the number of false star spots can be even larger than 10 times that of normal star spots;

(2) Complex optical environments, such as stray light from the Earth, Sun, and Moon, or scattered light from other satellite components, can significantly reduce the detection performance of star spots. For instance, as shown in Figure 5a, when the Moon enters the lower-left part of the field of view, multiple specular reflections form a “ghost image” in the upper-right part of the field of view, thus causing the global Constant False Alarm Rate (CFAR) threshold detection to fail;

(3) Further, there is certain degradation of the optical system and detector’s performance. Due to differences in thermal and mechanical environments, the degradation of an optical system manifests in that the star spots are no longer approximately Gaussian spots but have various morphologies, as illustrated in Figure 5c, which introduces difficulties to positioning. However, the degradation of the detector manifests in the appearance of a large number of dead pixels.

Nevertheless, the key to overcoming the aforementioned problems is the development of robust star spot detection algorithms and star identification methods. In view of that, at the end of this section, the collaborative processing steps of the star spot detection algorithm and SPPM-ADT are listed.

Consider Figure 5a, where a star sensor image with the Moon entering the field of view and forming a “ghost image” is presented. Then, the specific processing steps are as follows:

(1) Since the global CFAR threshold detection method fails, the Fast Minimum Bounding-Box Filter (FMBBF) [30] is employed for filtering. Then, run-length encoding is performed to connected domain, and star spots with fewer than 2 × 2 pixel are removed. Finally, 29 star spots are obtained, sorted from brightest to darkest, and numbered as No01–No29, as shown in Figure 5b;

(2) If the star sensor passes through the SAA, trajectory association of star spots between two consecutive frames is conducted to remove false star spots. In the case considered here, this step is omitted;

(3) Due to optical system degradation where star spots no longer approximate Gaussian profiles but exhibit diverse morphologies, as displayed in Figure 5c, the SPPM-ADT parameter is set as a star spot extraction error $\sigma_{S}$= 1.0 pixel;

(4) Next, the SPPM-ADT is used to identify star pairs, calculating the coarse attitude of the star sensor, as shown in Figure 5d;

(5) Based on the coarse attitude, 15 stars from the SAO star catalog’s local celestial sphere are projected onto the image plane, as depicted in Figure 5d;

(6) Afterward, the nearest neighbor matching process completes the star map recognition for all 13 identified stars, as shown in Figure 5e;

(7) Finally, using all identified stars from the star catalog, the star sensor’s attitude quaternion is calculated as ($q_{0}$, $q_{1}$, $q_{2}$, $q_{3}$) = (0.54690934, 0.18779778, −0.57585736, −0.57793639), where $q_{0}$ is the scalar component. When expressed in Euler angles 3-1-3, the attitude angles are (α, δ, ϕ) = (151.4821°, 15.4407°, 25.3579°).

Steps (1) and (2) represent single- and multi-frame star spot detection, respectively, and Steps (3)–(6) constitute the SPPM-ADT. However, the simulation analysis has shown that a robust detection algorithm can reduce the SPPM-ADT’s computation time and enhance identification probability. In addition, the relative independence of the SPPM-ADT from the star spot detection can provide a foundation for conducting simulation experiments.

## 5. Conclusions

This paper proposes an all-sky star identification method named SPPM-ADT. The proposed algorithm first rotates the guide star to become a polar star and uses the polar angle and azimuth angle of neighboring stars as polar pattern elements of the guide star. Also, the relative azimuth histogram is used for the spherical polar pattern matching, and a star pair after spherical polar pattern matching is achieved through angular distance cross-verification. Finally, a reference star image is generated using the identified star pair, and the matching of all guide stars in the field of view is realized. This study implements some optimization methods into the proposed SPPM-ADT algorithm, such as using Boolean addition instead of loop judgment, dynamically updating the matching threshold, terminating the loop matching in advance, and ranking guide stars and star spots according to brightness, thus reducing the identification time by two orders of magnitude. Further, the pattern star table only contains the compressed spherical polar pattern elements, and the star database capacity is small. The angular distance cross-verification of the star pair and star projection testing make the proposed SPPM-ADT algorithm insensitive to false and missing stars.

The results of the simulation experiments show that the proposed SPPM-ADT algorithm outperforms similar existing algorithms in terms of algorithm robustness, complexity, and database.

Due to differences between the ground and space environments and hardware degradation, star sensors might experience abnormal working conditions. Therefore, future work could focus on star identification under abnormal working conditions.

## Figures and Tables

**Table 1:** Typical medium field-of-view star sensors.

Parameter	Value
FOV	15° × 15°
Focal length	42.7793 mm
Resolution	1024 × 1024 pixel
Principal point	(512, 512)
Limiting magnitude	6.0 Mv
Standard deviation of position	≤5.0 pixel
Standard deviation of magnitude	≤1.0 Mv
Simulation image magnitude	≤9.0 Mv

**Table 2:** The SPPM-ADT database of the star sensor used in the simulation.

Table Type	Data Type	Number	Size (KB)
Region hash table	Unsigned short	450	0.88
Brightness ranking hash table	Unsigned short	4224	8.25
Spherical polar pattern hash table	Unsigned short	4224	8.25
Celestial coordinate table	Float	4224 × 2	33.00
Spherical polar pattern table	Unsigned char	56,449 × 2	110.25
Total			160.64

**Table 3:** The performance comparison of the SRPM and SPPM-ADT algorithms.

Algorithm	CPU	σ_S_ (pixel)	σ_Mv_ (Mv)	t_D_ (ms)	p_D_ (%)
SRPM	Intel i5-3210M	6.5	0.4	15	97.31
SRPM	Intel i5-3210M	8.0	0.4	22	85
SRPM	Intel i5-3210M	2.5	0.5	9.2	99.8
SRPM	Intel i5-3210M	2.5	1.0	18	88
SPPM-ADT	Intel i7-4790	6.5	0.4	0.52	99.69
SPPM-ADT	Intel i7-4790	8.0	0.4	0.71	99.41
SPPM-ADT	Intel i7-4790	2.5	0.5	0.28	99.91
SPPM-ADT	Intel i7-4790	2.5	1.0	0.53	98.95

**Table 4:** The performance comparison of the RRPM and SPPM-ADT algorithms.

Algorithm	CPU	σ_S_ (pixel)	σ_Mv_ (Mv)	t_D_ (ms)	p_D_ (%)
RRPM	Intel i3-2350M	1.0	0.4	1.80	99.91
RRPM	Intel i3-2350M	4.0	0.4	3.75	98.63
RRPM	Intel i3-2350M	1.0	1.0	4.73	95.45
SPPM-ADT	Intel i7-4790	1.0	0.4	0.15	100
SPPM-ADT	Intel i7-4790	4.0	0.4	0.30	99.95
SPPM-ADT	Intel i7-4790	1.0	1.0	0.33	99.89

**Table 5:** The performance comparison of the RTMM and SPPM-ADT algorithms.

Algorithm	CPU	σ_S_ (pixel)	σ_Mv_ (Mv)	t_D_ (ms)	p_D_ (%)
RTMM	Intel i5-11600K	2.0	0.3	10	98.41
RTMM	Intel i5-11600K	0.4	1.2	6	98.51
SPPM-ADT	Intel i7-4790	2.0	0.3	0.27	99.96
SPPM-ADT	Intel i7-4790	0.4	1.2	0.52	99.44

**Table 6:** The performance comparison of the ADMST and SPPM-ADT algorithms.

Algorithm	CPU	σ_S_ (pixel)	σ_Mv_ (Mv)	t_D_ (ms)	p_D_ (%)
ADMST	Intel i5-12490F	5.0	0.4	12	99.8
ADMST	Intel i5-12490F	1.0	0.9	13	97.5
ADMST	Intel i5-12490F	1.0	1.5	18	96
SPPM-ADT	Intel i7-4790	5.0	0.4	0.39	99.78
SPPM-ADT	Intel i7-4790	1.0	0.9	0.38	99.60
SPPM-ADT	Intel i7-4790	1.0	1.5	1.18	97.62

## Data Availability

All code, data, and materials included in this research are available upon request by contact with the corresponding author.
